# Recent Advances in Polymeric Nanocomposites of Metal-Organic Frameworks (MOFs)

**DOI:** 10.3390/polym11101627

**Published:** 2019-10-09

**Authors:** Jun Zhong, Ranjith Kumar Kankala, Shi-Bin Wang, Ai-Zheng Chen

**Affiliations:** 1 Institute of Biomaterials and Tissue Engineering, Huaqiao University, Xiamen 361021, China; 18013087045@hqu.edu.cn (J.Z.); sbwang@hqu.edu.cn (S.-B.W.); 2 College of Chemical Engineering Huaqiao University, Xiamen 361021, China; 3 Fujian Provincial Key Laboratory of Biochemical Technology (Huaqiao University), Xiamen 361021, China

**Keywords:** metal-organic frameworks, porosity, biocompatibility, surface functionalization, bio-interfaces

## Abstract

Recently, metal-organic frameworks (MOFs) have garnered enormous attention from researchers owing to their superior physicochemical properties, which are of particular interest in various fields such as catalysis and the diverse areas of biomedicine. Despite their position in the utilization for various applications compared to other innovative nanocarriers such as dendrimers and mesoporous silica nanoparticles (MSNs), in terms of advantageous physicochemical attributes, as well as attractive textural properties, ease of characterization, and abundant surface chemistry for functionalization and other benefits, MOFs yet suffer from several issues such as poor degradability, which might lead to accumulation-induced biocompatibility risk. In addition, some of the MOFs suffer from a shortcoming of poor colloidal stability in the aqueous solution, hindering their applicability in diverse biomedical fields. To address these limitations, several advancements have been made to fabricate polymeric nanocomposites of MOFs for their utility in various biomedical fields. In this review, we aim to provide a brief emphasis on various organic polymers used for coating over MOFs to improve their physicochemical attributes considering a series of recently reported intriguing studies. Finally, we summarize with perspectives.

## 1. Introduction

In recent years, metal-organic frameworks (MOFs), a new class of highly porous architectures, have garnered enormous interest from researchers, owing to their attractive physicochemical properties and advantageous morphological attributes [1]. Notably, the large-sized interior cavity of these MOFs resulting in an open and interconnected porous network was of particular interest initially in several applications such as adsorption [2,3,4,5,6]. Further, the adsorption properties of MOFs, coupled with the catalytic activity of their functional units at the ligand and second building unit (SBU) active metal sites, have led to potential interest as highly functional porous materials in catalysis [7,8]. In this context, researchers have also succeeded in increasing the maximum pore size within the MOF to 10 nm, which could be highly feasible to be applied towards biomedical applications in terms of allowing the carrying ability of larger biomolecules. These chronological advancements have gradually further opened up new dimensions of MOFs towards diverse biomedical applications such as drug delivery, and bioimaging, among others [9,10,11].

Broadly speaking, based on the chemical composition and intrinsic physicochemical attributes, nanobiomaterials can be categorized into three different classes: organic nanosystems (liposomes, dendrimers, micelles, and polymeric constructs); inorganic nanoparticles (quantum dots, noble metal nanoparticles, MNPs, iron oxide nanoparticles, silica nanoparticles, and upconverting nanoparticles, UCNPs); and organic-inorganic hybrid composites [12,13,14,15,16,17,18]. Comparatively, organic matter-based materials are considered for biomedical applications over inorganic nanomaterials owing to their relatively higher biodegradability and compatibility [19,20]. However, they suffer from significant limitations of inherent instability and poor encapsulation efficiency issues, which limited their applicability. On the other hand, inorganic-based materials could address these intrinsic limitations of organic materials and also offer specific characteristics such as magnetism, autofluorescence ability, and plasmonic resonance, among others [21,22,23,24]. However, the intrinsic stability of such inorganic-based materials often results in their low biodegradation rate, leading to long-term accumulation-induced biosafety risk. In fact, inorganic nanoconstructs require an organic surface coating to ensure the stability of the colloid, similar to biological media, as various organic molecules such as plasma proteins are inevitably adsorbed onto the surface of inorganic nanoconstructs to substantially enhance their compatibility and extend their circulation half-life in vivo [25,26]. Thus, tremendous attention among researchers in the fabrication and utilization of these innovative organic-inorganic hybrid nanobiomaterials continues to rise.

MOFs are of such novel classes of hybrid nanobiomaterials composed of covalently-linked organic building units (such as organic linkers) to the nano-sized inorganic units, such as metal oxide clusters [27,28]. Therefore, MOFs exhibit all the intrinsic properties of materials at the nanometer level, in addition to the large specific surface area and high porosity of bulk MOF, which are of particular interest in diverse applications such as adsorption [2,3,4,5,6,29,30,31], filtration [32], catalysis [7,8], and various biomedical applications [33,34,35,36]. Typically, various metal ions (di-, tri-, tetravalent, and others) can be used. In general, MOFs are fabricated by using some of the traditional methods based on solvothermal or nonsolvothermal strategies. These approaches could result in the fabrication of MOFs in different geometries, such as linear, square planar, and triangular, among others, based on both the inorganic cluster and organic linkers. Owing to their diversity, it is highly feasible to fabricate a large number of different structures. However, these approaches could not meet the on-demand requirements for industrial production since they are time consuming and expensive. Thus, these issues prompted researchers to seek new ways for synthesizing MOFs [37], such as microwave-assisted synthesis [38], sonochemical synthesis [39], electrochemical synthesis [40], and mechanochemical synthesis [41]. Since MOFs are composed of metal species or clusters of diverse organic ligands, the structure of MOF is determined by the organic ligands for a certain class of metal-based MOFs [42], such as ditopic carboxylate linkers [43], tritopic carboxylate linkers [44], tetratopic carboxylate linkers [45], hexatopic carboxylate linkers [46], octatopic carboxylate linkers [47], desymmetrized linkers [48], and *N*-heterocyclic linkers [49]. Selecting different metal ions and organic linkers, can endow MOFs for various applications, such as stimuli-responsiveness in drug delivery [50], toward pH-responsive [51], magnetic-responsive [52], molecular-responsive [53], thermos-responsive [54], and pressure-responsive MOFs [55]. Compared to widely utilized polymeric dendrimers, and mesoporous silica-based materials for drug delivery application, the crystalline MOF structures have better reproducibility, drug loading, and release characteristics due to ordered and uniform-sized porous architectures [56,57,58,59,60,61,62,63,64,65,66,67,68,69,70,71,72,73,74,75,76]. Owing to their peculiar textural properties of chemically-tunable large pore sizes, MOFs have attracted enormous interest as drug carriers in conveying diverse therapeutic molecules in their interior frameworks [77,78,79]. More often, these carriers suffer from poor loading efficiency of drugs. In most of the instances, these guest species are encapsulated within the MOF channels with physical interactions or electrostatic interactions, which are significantly unstable during drug delivery. Moreover, the difference in the concentration of the drug within the carrier and the solution boosts their leakage from the frameworks [80,81]. In addition to poor encapsulation efficiency, this could lead to premature release of the drug molecules during delivery in vivo, which is highly challenging for their application in the field of biomedicine [82,83].

**Figure 1:**
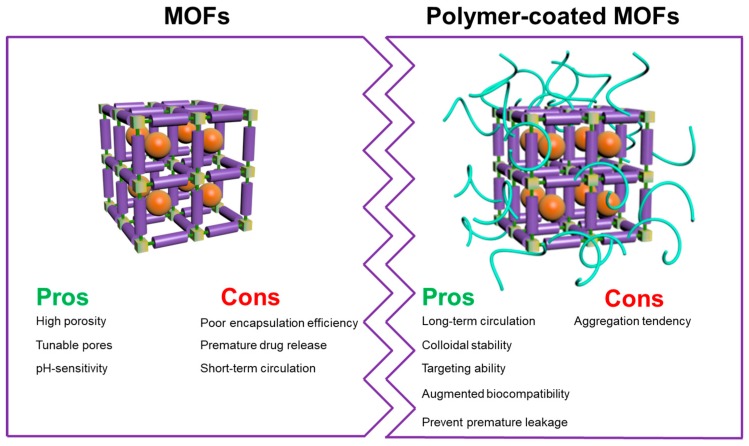
Schematic illustration the combination of polymer and MOF and their pros and cons.

In order to utilize MOFs as a drug-delivery platform, many research groups are currently aiming at addressing the compatibility, stability, and degradability of the carrier in the biological environment, and enabling the drug carrier to have an excellent distribution in vivo [84,85,86,87,88]. However, for different MOFs, metal ions and organic ligands are randomly combined, which yield different physicochemical attributes, some of which can be in a stable state for a certain period of time [89,90,91,92,93]. Under physiological conditions, the instability of MOFs can be addressed by external surface coating with polymer to endow them with unique properties for drug delivery. Moreover, the thickness of the coating can be further increased, allowing the nanocarriers to convey the guest species appropriately to the target site prior to their degradation [94,95,96,97,98,99,100,101,102,103,104,105,106,107,108,109,110,111]. In this review, we emphasize recent advances in fabricating various MOFs with organic polymers to improve their physicochemical attributes considering a series of recently reported intriguing studies, as shown in Figure 1. Although there exist numerous types of MOFs based on composition, porosity, stability, and various physicochemical attributes, it should be noted that this article highlights the advances of various predominantly chosen MOFs, such as ZIFs, MILs, UiOs, and PCNs, among others, for biomedical applications.

## 2. Surface-Coated MOFs

As mentioned earlier, the highly ordered textural properties of MOFs allow them to conveniently encapsulate the therapeutic guest species in the interior, implying their potential for utilization in the field of drug delivery [77,78,79]. However, some of the MOFs suffer from stability issues in the aqueous solution, leading to premature drug release, which hinders their applicability as a drug carrier [80,81,82,83]. Functional polymer coating over MOFs is an effective method to address various limitations of MOFs such as poor biocompatibility, instability, short circulation time, and rapid degradability. Many polymers, like chitosan [112], heparin [113], poly(N-vinylpyrrolidone) (PVP) [114], and poly(sodium 4-styrenesulfonate) (PSS) [115], among others, having excellent aqueous solubility and biocompatibility, could be wrapped on the surface of MOFs. Since MOFs are composed of various multivalent metal species and organic linkers, polymers possessing functional groups such as –C=O, –COOH, could offer coordination interactions with such metal species in MOFs [36]. These polymer-coated MOFs provide several advantages such as long circulation time, avoiding premature leakage of drugs, increased colloidal stability in physiological buffers with high ionic strength, augmented biodistribution characteristics, anti-macrophage clearance, and controlled binding efficiency with the proteins in the biological environment [116]. In addition, other specific advantages of the polymer-coated MOFs in terms of therapeutic benefits include precise uptake by cancer cells over normal cells through specific interactions by the surface coated polymers with the overexpressed proteins on the cancer cell surfaces [117]. Various other benefits comprise of specific shielding effects against harsh biological environment, ease of integrating targeting as well as bioreactive domains over the surface could enhance the therapeutic efficiency of the MOFs through appropriate delivery of guest species at the target site [114].

**Figure 2:**
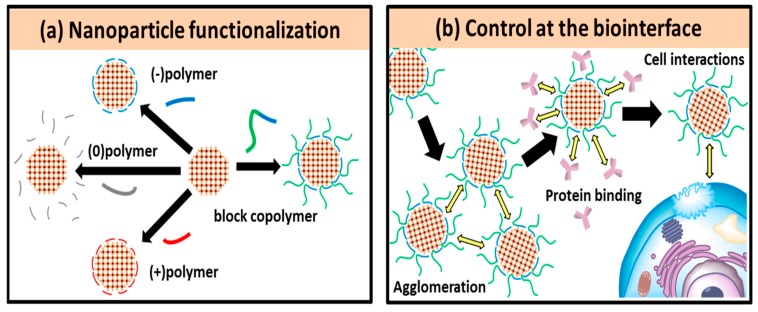
(**a**) Schematic illustrating the coating procedure for four different polymer groups; (**b**) Studies with functionalized MOF biointerfaces. Reproduced with permission from Ref. [36] Copyright 2019, American Chemical Society.

In this framework, nano-sized MOFs can be coated with different polymers by functionalizing their surfaces with covalent, as well as coordination linkages. In a case, Zimpel and coworkers [36] proposed a method for establishing coordinating linkages of polymers on the surface of MOFs. As depicted in Figure 2, the Zr-fum nanoparticles were synthesized using the precursors, ZrCl_4_, fumaric acid, and formic acid. The authors proposed that different polymers could compete with formic acid based on the principle of increase in the entropy. Further, these polymers after coordination were attached to the outer surface of the MOFs. In this context, the coating procedure was investigated using four different polymer groups of negatively- as well as positively-charged, neutral, and hybrid block copolymer) and further explored the interactions between the functionalized MOFs and biological interfaces, including aggregation, and interactions with membrane and substantial binding to cell surface proteins. Evidently, no changes in the shape as well as position of the corresponding peaks of Zr-fum after coating with different polymers, indicating that the polymers were bound efficiently to the MOF surface. Moreover, no effect on the crystalline nature of MOF, and no changes in the morphology of Zr-fum MOFs after coating with polymers were observed, attributing to the stable frameworks of MOFs. The authors claimed that the method of polymer functionalization through coordination interactions could be applied to other MOFs in fabricating highly efficient polymeric nanocomposites. In this section, we discuss regarding various polymer-coated MOFs for their utilization in diverse biomedical fields, highlighting the pros and cons. Different approaches for synthesizing polymer-coated MOFs and their diversified advanced biomedical applications are listed Table 1.

### 2.1. Chitosan

Chitosan, extracted from crustacean shells, is a polysaccharide that is predominantly used in biomedical applications owing to its degradability and compatibility [118]. In addition, it has been marketed as a binder in the cholesterol-lowering formulations [119]. The pH-responsive nature of this promising biopolymer, i.e., dissolution in the acidic pH, has garnered enormous attention from researchers in its utilization in diverse biomedical fields [120]. Biocompatible MOFs nanoparticles (nanoMOFs) are ideal pharmaceutical carriers for drug delivery, owing to their intrinsic stability and small size facilitating cross biological barriers [77,78,79]. To date, most of the reported studies have focused only on their administration to the intravenous route involving pain and serious complications. However, the most convenient oral administration of nanomembranes are often disregarded due to multiple biological barriers resulting in the poor bioavailability of delivered drugs [121]. In an attempt to address this issue, Hidalgo and colleagues [112] proposed a bio-friendly nanocarrier based on biocompatible mesoporous iron (III) trimethylsulfonate nanoparticles coated with bio-adhesive polysaccharide chitosan for oral drug delivery applications. This method of administration has shown no effect on the structural properties and adsorption/release capabilities of nanocomposites owing to their surface engineering with the biopolymer chitosan. The interactions between the MOF surface and chitosan were systematically characterized by computational simulation and high-resolution soft X-ray absorption techniques. Further, the effects of chitosan coating on the colloidal and chemical stabilities under oral simulation conditions were confirmed. Finally, the biocompatibility, as well as the intestinal barrier bypass ability of these chitosan-coated MOFs were evaluated in vitro, which resulted in higher intestinal permeability compared to uncoated materials, maintaining optimal biocompatibility. Moreover, the chitosan-coated nanoparticles offer excellent physicochemical properties, good colloidal stability and biodegradability, and good intestinal barrier bypass function, which make these nanocomposites promising nanocarriers for drug delivery through oral administration [112].

### 2.2. Heparin

**Figure 3:**
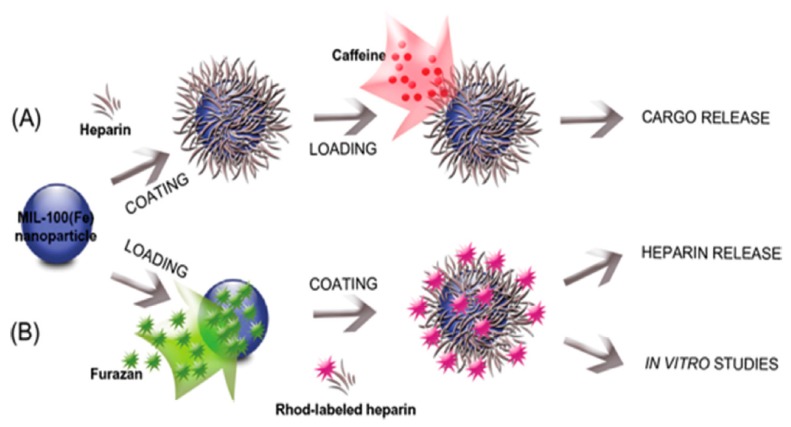
Schematic illustration of the formation of heparin-coated MIL-100(Fe) nanoparticles loaded with specific cargoes via two different pathways: (**A**) Heparin is selectively coated on the external surface of the NP followed by the post loading with caffeine; and (**B**) preloading with furazan followed by external coating of the NP with rhodamine-labeled heparin. Reproduced with permission from Ref. [113] Copyright 2015, John Wiley & sons.

Heparin is one of the naturally occurring sulfate-rich polysaccharides used in diverse biomedical fields [122]. Currently, there are two main forms of heparin in clinical use, including unseparated heparin and low Mol. Wt. heparin. The average Mol. Wt. of unseparated heparin ranges from 3–30 kDa, while the low Mol. Wt. heparin ranges from 4–5 kDa [123]. Although it is highly convenient to encapsulate MOFs using various polysaccharides, it is highly challenging to modify the outer surface of nanoMOFs without changing the porous architectures of MOFs. Elena and colleagues [113] reported a method for functionalizing the surface of MIL-100 (Fe)-based MOFs without altering their porous architectures. As shown in Figure 3, the fabricated MIL-100 (Fe) MOFs were coated with heparin on their surface, using the hydrophilic functionality of heparin to prolong their circulation time in the body. The final particle size of uncoated MIL-100(Fe) was varied with the dispersion media, resulting in 141 ± 13, 155 ± 61, and 162 ± 60 nm in water, PBS, and PBS + albumin mixture dispersion media, respectively. Further, two different approaches were used for fabricating heparin-coated MIL-100(Fe) nanoparticles. In one of the methods, heparin was selectively coated initially on the surface of MOFs, and then loaded with caffeine. In another approach, MOFs were preloaded with furazan and then coated with rhodamine-labeled heparin on their surface. Remarkably, after encapsulation with heparin, the original crystal structure and porosity of MOFs were remained uninterrupted. Moreover, these polymer-wrapped MOFs offered controlled release ability of encapsulated guest species. In addition, the colloidal stability of these functional nanocomposites was significantly improved. These experimental results revealed that the coating of polymers over the surface of a nanofilm using a direct method could augment the versatility, thereby increasing their potential of highly porous MOFs in the biomedical field.

### 2.3. Hyaluronic acid

Hyaluronic acid (HA), a non-sulfated glycosaminoglycan polysaccharide composed of β-1,4-D-glucuronic acid-β-1,3-N-acetyl-D-glucosamine disaccharide units linearly, is widely distributed in the body such as eye vitreous and extracellular matrix (ECM) of the cartilage tissue [124]. HA (Mol. Wt. of 100 to 8000 kDa), a highly hydrophilic polymer macromolecule, is capable of binding to a large amount of water [125]. This attractive property of HA can prolong the circulation time of the encapsulated nanoparticles in the blood and increase their drug delivery efficiency. Moreover, HA coating enhances the stability of the drug carrier in solution, ensuring its safety before reaching the tumor site. In addition, HA acts as a targeting agent, allowing the drug carrier to target the tumor site, not only improving the drug delivery at the target site, but also prevents the drug carrier from being entry to normal cells, resulting in reduced adverse effects. Moreover, HA provides a molecular-responsive release function for the drug carrier as the internalized MOF@HA can be degraded by the hyaluronidase enzyme, resulting in the release of the drug specifically in the intracellular microenvironment.

**Figure 4:**
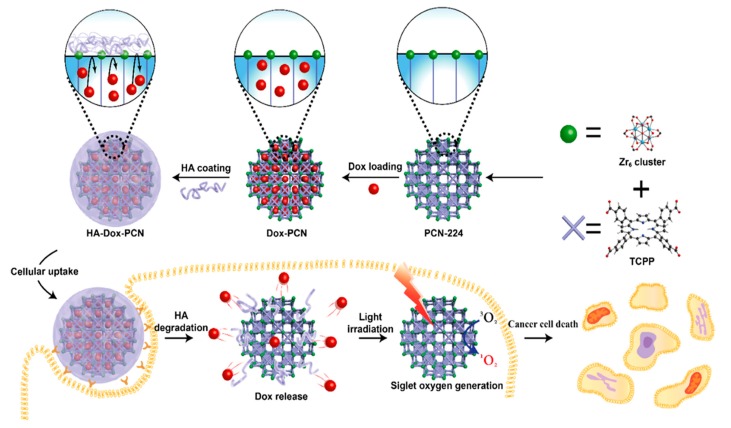
Illustration showing the preparation of HA as a gate keeper in MOF-based nanosystems for chemo- and PDT-combined therapy. Reproduced with permission from Ref. [126] Copyright 2019, American Chemical Society.

To demonstrate these interesting attributes of HA-coated MOFs, Kim and colleagues [126] reported a method of wrapping drug species-encapsulated MOFs with HA to prevent the premature release of drug molecules and substantial efficient delivery at the target site. As shown in Figure 4, Zr^4+^ as a metal source and photosensitizer tetrakis(4-carboxyphenyl) porphyrin (TCPP) as organic linkers were initially used to fabricate PCN-224-based MOFs with large-sized pores through coordination between them. Further, the anticancer drug doxorubicin (DOX) was doped within the pores of these MOFs through electrostatic adsorption, and the surface was then coated with a layer of HA, for limiting the diffusion of encapsulated DOX molecules from the MOF channels. Herein, the HA on the surface not only effectively prevented the early release of the drug but also facilitated the targeting of specifically overexpressed HA receptor on cancer cells, which significantly enriched the internalization efficiency of MOFs into cancer cells. The internalized HA-encapsulated MOFs significantly delivered DOX species intracellularly after when the hyaluronidase enzyme degraded the surface HA, promoting the MOFs exposure to cytosol. Further, TCPP absorbed the irradiated light at a specific wavelength effectively converted O_2_ to deadly singlet oxygen (^1^O_2_) species, which resulted in substantial ablation of cancer cells by interacting with mitochondria DNA thereby enabling cell apoptosis. Using the combinatorial strategy of chemotherapy and photodynamic therapy (PDT), it could be highly convenient to significantly ablate cancer cells.

Compared to other porous materials such as MSNs, MOFs could offer numerous advantages for their application in drug delivery [127]. Considering the example of HA-coated composites, on one hand, in addition to targeting ability, some of the organic ligands in MOFs (such as TCPP) could convert O_2_ to ^1^O_2_ and participated in synergistic tumor ablation effects while the MSN frameworks could only encapsulate and delivery the therapeutic guest species. Further, these composites (both the coated HA and the core MOF) could be degraded in the physiological environment and the degraded products could be expediently eliminated from the body. On the other hand, when compared with other advanced metal oxide, such as MnO_2_, the MOFs coated with polymers could provide additional benefits in terms of degradability and compatibility attributes. In an example, Min et al. [128] encapsulated MOFs within the MnO_2_ shell for biomedical applications. MOFs had shown no significant toxic effects on 4T1 cells with >95% viability at a concentration of 50 µg/mL, while the MOFs coated with a layer of MnO_2_ resulted in reduced viability of 4T1 cells at a similar concentration. Further attempts of cell membrane coating were made to augment the compatibility. However, the viability of cells was relatively incomparable to pure MOFs. In another case, Hidalgo and colleagues [112] coated MIL-100 (Fe) with chitosan polymer, which resulted in excellent biocompatibility with viability greater than 95%, even at the concentration of 1200 µg/mL. These findings explicitly clarify that the polymer-coated composites are highly compatible and such advancements are highly beneficial over others for biomedical applications (Table 1).

### 2.4. PVP

**Figure 5:**
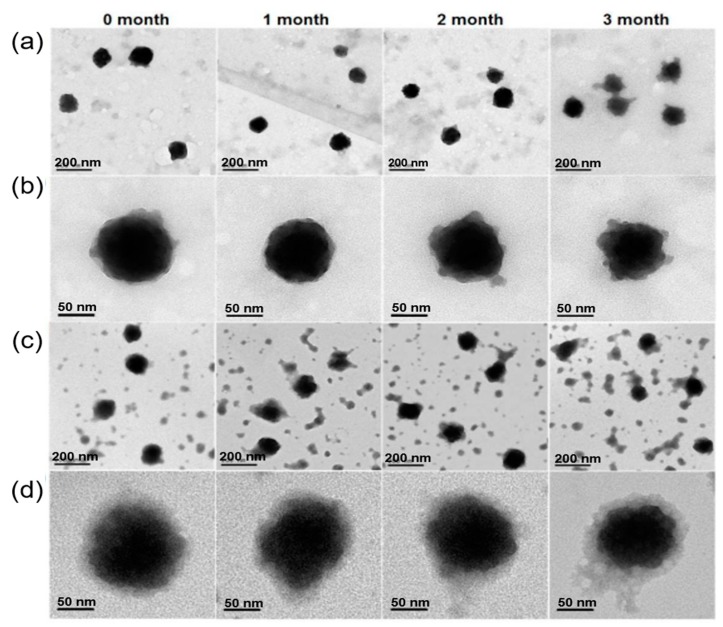
Long-term stability of PVP-coated BSA@ZIF-8 nanoparticles. (**a**) TEM images of PVP-coated BSA@ZIF-8 nanoparticles in PBS at different times; (**b**) High-resolution TEM images of PVP-coated BSA@ZIF-8 nanoparticles in PBS at different times; (**c**) TEM images of PVP-coated BSA@ZIF-8 nanoparticles in human serum (10%) at different times; (**d**) High-resolution TEM images of PVP-coated BSA@ZIF-8 nanoparticles in human serum (10%) at different time. Reproduced with permission from Ref. [130] Copyright 2018, American Chemical Society.

PVP is an amphiphilic, non-ionic polymer synthesized using acetylene [129]. PVP shows excellent physicochemical properties, such as excellent aqueous solubility, low toxicity, and good chemical stability, which are of particular interest in diverse biomedical applications [114]. This polymer can be used as a surfactant for stabilizing diverse nanoparticles in the polar solvents, and also as a capping agent in controlling their morphology (size and shape) during fabrication. In an attempt to encapsulate MOFs in PVP, Chen and colleagues [130] designed highly stable nanoparticles by modifying the surface of BSA@ZIF-8 MOFs by coating with biocompatible PVP. Further, the authors investigated the stability of nanoMOFs for the first time in cell media supplemented with 10% fetal bovine serum for more than 3 months ensuring the long-term stability of such PVP-based nanocomposites in cellular media for their application in biomedical field. From the scanning electron microscopy (SEM) observations (Figure 5), it could be observed that no severe aggregation and changes in the final particle sizes of BSA@ZIF-8 were observed, attributing to the stability of PVP coating over the designed MOFs in the culture media.

**Figure 6:**
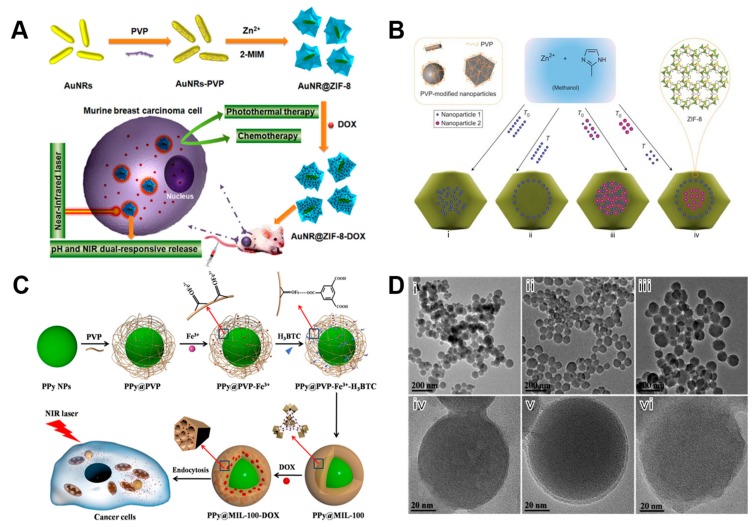
(**A**) Strategy for fabrication of AuNR@ZIF-8 core-shell nanostructures as a novel multifunctional nanoplatform for synergistic chemo-photothermal cancer therapy both in vitro and in vivo. Reproduced with permission from Ref. [131] Copyright 2018, Springer Nature. (**B**) Scheme of the controlled encapsulation of nanoparticles in ZIF-8 crystals. The spatial distribution of incorporated PVP-modified nanoparticles within ZIF-8 crystals can also be controlled by their addition sequence (that is, addition at the beginning (T0) or after a certain time (T) during the MOF synthesis). Reproduced with permission from Ref. [132] Copyright 2012, Nature publishing group. (**C**) Schematic illustration of multifunctional nanoparticles consisting of a PPy core and a mesoporous MIL-100 shell, designed for simultaneous PTT and chemotherapy of cancer cells. (**D**) TEM images of PPy nanoparticles (i and iv), of PPy@MIL-100 for 1.5 h of reaction time (ii and v), and of PPy@MIL-100 for 24 h of reaction time (iii and vi). Reproduced with permission from Ref. [133] Copyright 2016, American Chemical Society.

In addition to coating over MOFs, PVP can be used as surfactant or a structure-directing agent, to coat over the core materials for stabilizing the shell structures. This polymer predominantly offers a significant advantage of biocompatibility over other stabilizers such as CTAB, which is of particular interest in the biomedical applications. Indeed, numerous conventional therapies have been used so far for eradicating several dreadful diseases like cancer, including radiation therapy, surgery, immunotherapy and chemotherapy. Recently, several progressions have been made in developing various advanced therapeutic strategies by formulating numerous designs that could exhibit multiple effects using a single therapy and by substantially avoiding side effects. In this framework, several metal nanoparticles-based core-shell structures have been fabricated for improving the therapeutic efficiency of the formulation. In a certain case, Li and colleagues [131] synthesized AuNR@ZIF-8-based core-shell nanoarchitectures (Figure 6A). Initially, cetyltrimethylammonium bromide (CTAB)-stabilized gold nanorods (AuNRs) were prepared. Subsequently, considering the potential toxicity of cationic CTAB to cells and the excellent binding ability of PVP to AuNRs, the stabilizer CTAB molecules on the surface of AuNRs were exchanged with PVP. Then, 2-MIM (2-methyl imidazole) was rigorously stirred with PVP-stabilized AuNRs. Further, the aqueous Zn(II) solution was added to the mixture in methanol, resulting in the AuNR@ZIF-8 nanocomposites. These innovative MOF-based core–shell nanoarchitectures have shown synergistic chemo-photothermal therapy.

Indeed, PVP can be used as a stabilizer during the fabrication of nanomaterials to control the morphological attributes of the nanoconstructs [134]. Interestingly, Lu and coworkers [132] proposed an innovative controlled encapsulation strategy, for the fabrication of various hierarchical architectures based on PVP-coated nanoparticles with different sizes and shapes, which were substantially wrapped with a layer of ZIF-8 on their surface (Figure 6B). In this work, they showed that different shapes of PVP-modified nanoparticles could be wrapped in MOF in a well-dispersed fashion mediated by the coordination bonds between PVP and zinc ions. In addition, the non-polar component in PVP could be bound to the organic ligand of MOF by the hydrophobic interactions. In another case, Zhu and coworkers [133] used PVP as a template to promote the self-assembly of Fe^3+^ ions and H_3_BTC. The surface of polypyrrole (PPy) nanoparticles was initially modified with PVP to enhance their stability and substantially augment the binding efficiency of the precursor of MIL-100 (Figure 6C). On one end, Fe^3+^ remaining on PPy could be used as a metal source for the synthesis of MIL-100. To this end, PVP adsorbed on the surface of PPy could enhance the affinity of nanoparticles with MIL-100 precursor by weak coordination interaction between pyrrolidone ring (C=O) and Fe^3+^ of PVP. The polypyrrole reacted with the MIL-100 precursor to form a shell structure, indicating the formation of PPy@MIL-100 nanocomposites with a core-shell structure. From Figure 6D, the fabricated nanoparticles have shown a thin layer of shell on the surface, and the shell thickness was gradually increased with the coating time. Similarly, Deng and colleagues [135] fabricated core-shell nanoarchitectures based on the mesoporous MOF as a shell and the UCNPs as cores using the PVP as a stabilizer over UCNPs. The authors demonstrated that the PVP was uniformly distributed on UCNPs, resulting in the composites with an average size of 30 nm and excellent monodispersity. It should be noted that the particle size of UCNPs@MOF nanoclusters was increased with the increase of reaction time.

### 2.5. PSS

**Figure 7:**
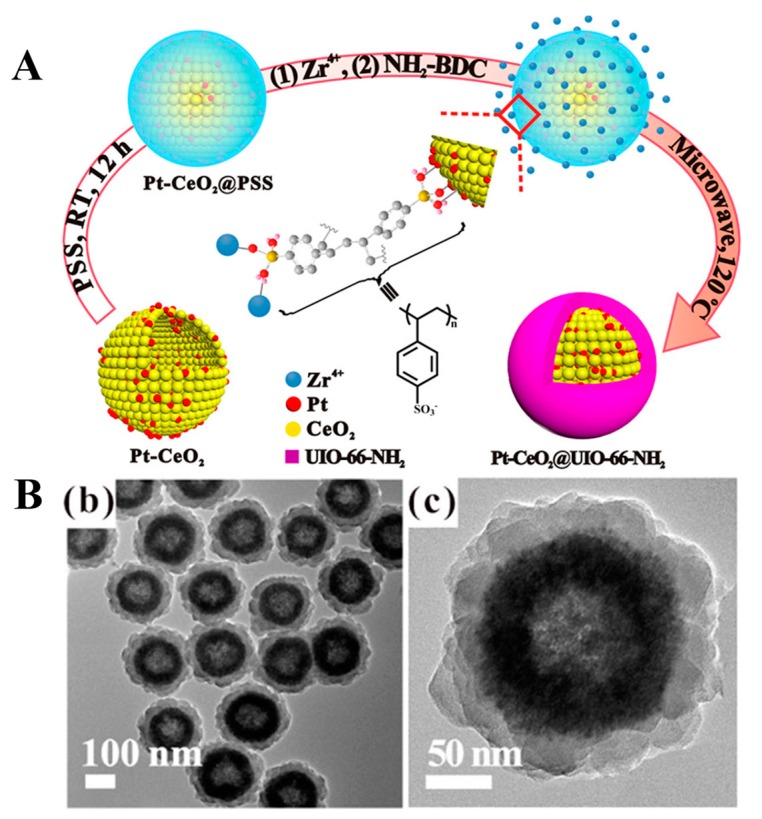
(**A**) Schematic Illustration for the Formation of PtCeO_2_@UiO-66-NH_2_. (**B**) TEM images of Pt-CeO_2_@UiO-66-NH_2_ (**b**) Scale bar 100 nm and (**c**) 50 nm. Reproduced with permission from Ref. [115] Copyright 2018, American Chemical Society.

PSS is another negatively-charged polymer, which can be used to coat over the surface of MOFs, enabling the ease of adsorption of cations from the environment through the electrostatic adsorption. More often, this ability of adsorption facilitates the convenient growth of MOFs in situ. Long and coworkers [115] used PSS to prepare a layer of Pt/CeO_2_ toward microwave-assisted fabrication of Pt-CeO_2_@MOF core-shell hybrid architectures. From Figure 7, the Pt-CeO_2_ nanoparticles were spherical in shape with a large hollow cavity. After the surface was wrapped with PSS, Zr^4+^ could be adsorbed, resulting in a uniform layer on the surface of Pt-CeO_2_, however, the thickness remained unchanged. Moreover, the utilization of PSS further played a critical role in the formation of complete UiO-66-NH_2_ shell over these Pt-CeO_2_@MOF structures. The formation of hierarchical structures was substantially confirmed using various characterization techniques. Interestingly, PSS acted as a connecter, which eventually reduced the interfacial energy between the Pt-CeO_2_ and UiO-66-NH_2_, thus promoting the growth of UiO-66-NH_2_ on Pt-CeO_2_ through electrostatic interactions.

**Figure 8:**
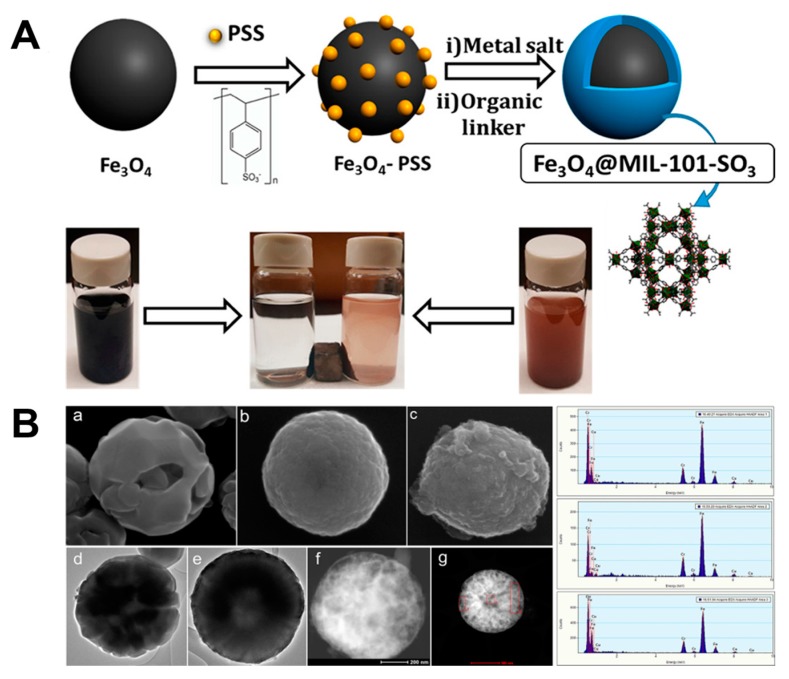
(**A**) Schematic representation of the Fe_3_O_4_@MIL-101-SO_3_ core formation. (**B**) SEM, Transmission Electron Microscope (TEM), and Energy Dispersive X-ray (EDX) studies of the magnetic core-shell microsphere. (**a**–**c**) SEM images of Fe_3_O_4_, Fe_3_O_4_–PSS, and Fe_3_O_4_@MIL-101-SO_3_. (**d**–**f**) TEM images of Fe_3_O_4_, Fe_3_O_4_–PSS, and Fe_3_O_4_@MIL-101-SO_3_. (**g**) TEM and corresponding EDX data showing elemental composition of Fe_3_O_4_@MIL-101-SO_3_. Reproduced with permission from Ref. [136] Copyright 2017, American Chemical Society.

In another case, Elsaidi and colleagues [136] synthesized microspheres with clear interface using Fe_3_O_4_ as the core and MIL-101-SO_3_ as the shell and subsequently functionalized these MOFs. The synthesized Fe_3_O_4_ microspheres were spherical and possessed a diameter of around 300 to 500 nm. In order to grow a MOF shell on the outer layer of Fe_3_O_4_, they added PSS as a linker. PSS can not only absorb metal ions, promoting the formation of MOF shell, but also provide sulfonic acid (SO_3_^−^) groups, which can balance the charge of Na^+^ or H^+^ cations [137]. These cations make material more stable in the aqueous solution [138], which broaden their application in biomedical applications (Table 1). After the MOF was wrapped on the surface of Fe_3_O_4_, the diameter of the microspheres increased to 800–900 nm, and its shape has not changed significantly, and remained spherical (Figure 8). Further, these stable magnetite@MOF composites were applied toward the extraction of rare earth elements [139]. Due to the uniform distribution of many negatively-charged sulfonic acid (SO^3-^) groups, these MIL-101-SO_3_ shells could efficiently exchange the rare earth ions in water. Interestingly, the magnetic property of the Fe_3_O_4_ core could facilitate their ease of separation from the aqueous solution mixture.

## 3. Cross Linking MOF to Polymer

**Figure 9:**
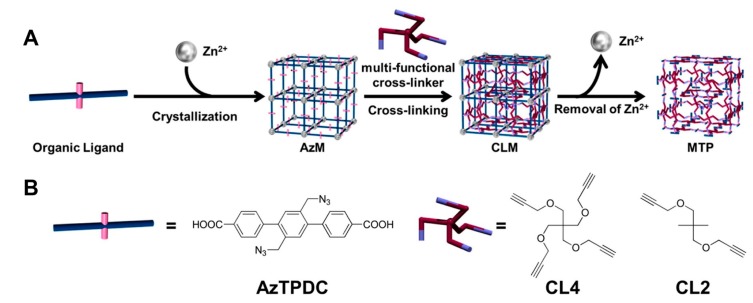
(**A**) Schematic illustration of cross-linking of the organic linkers in MOF (AzM) and subsequent decomposition to obtain polymer gel (PG). (**B**) Molecular structures of the organic ligand (AzTPDC) and the cross-linkers. Reproduced with permission from Ref. [145] Copyright 2013, American Chemical Society.

Except a few specific types of MOFs, such as Fe- and Zr-based MOFs, most of the MOF composites suffer from poor aqueous stability, which seriously hinders their application in diverse biomedical fields (Table 1) [143,144]. Although several advancements have been made through proposed ways to improve their aqueous stability, MOFs still suffer from this issue. Even the surface-coated polymers on MOFs through self-assembly (discussed in Section 2) have not solved this issue to a considerable extent. However, several efforts have been dedicated such as crosslinking the organic ligands of MOF, facilitating the formation of polymeric composite of 3D structure, which could essentially improve the stability of MOFs in the aqueous solution. In this context, Ishiwata and coworkers [145] transformed Zn-based MOFs to polymer gel (PG) using the cross-linking approach (Figure 9). Initially, azide-tagged MOF (AzM) structures were fabricated by the self-assembly of Zn^2+^ with the organic ligand diazide-triphenyldicarboxylic acid ligand (AzTPDC). Further, different crosslinking agents, such as tetra-acetylene cross-linker (CL4) and diacetylene cross-linker (CL2), were used, resulting in the cross-linked MOF (CLM) complexes. Finally, Zn^2+^ species were removed to obtain MOF-templated polymer (MTP). After removal of Zn^2+^, MTP became amorphous, indicating that the crystal structure of MOF was demolished and has transformed from a hard material to a soft PG material. Moreover, the colloidal particles or macromolecules in the solution were connected, resulting in a spatial network-like architecture. Moreover, the structural voids were filled with a liquid, and such a special dispersion system called as PG. Since the organic linkers in MTP were connected to each other, the water molecules could not disrupt the binding sites between the metal species and organic linkers. This could be the plausible reason for the aqueous stability of MTP, and cleared the barriers for the application of MTP in the biomedical field. Contrarily, in a case, Howarth and coworkers [146] demonstrated that the intermolecular interactions between the metal ions and organic ligands in MOFs were relatively weak. Such junctures could be hydrolyzed in the aqueous environment, resulting in their corresponding protonated linkers or hydroxide forms, and may eventually collapse the intrinsic structure of MOFs. Although the MOFs and polymer gel are totally two different materials with hard and soft in nature, respectively, and their conversion would be highly challenging, however, the MOFs were transformed to gels by simple crosslinking and removal of the metal species.

**Figure 10:**
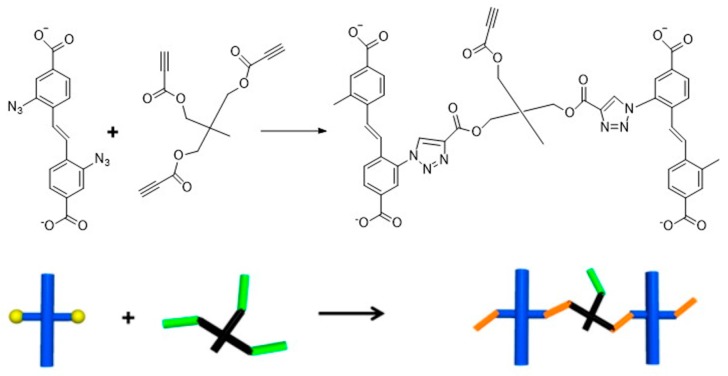
Schematic representation showing the copper-free click reaction between the diazido-stilbenedicarboxylic acid (DA-SBDC) and the cross-linker trimethylolethane tripropiolate. Reproduced with permission from Ref. [148] Copyright 2014, American Chemical Society.

The water-susceptible sites in MOFs are easy to be hydrolyzed in the aqueous solution, and facilitate the release of the metal ions, which may significantly affect the cell growth. Although researchers have made colossal efforts to reduce these effects, such as coating of polymer on MOFs [147]. However, it still remains unaddressed. Further, these consequences drive researchers in search for a metal-free way to synthesis a medium for biomedical applications. Tsotsalas and colleagues [148] synthesized a copper-free polymer gel with excellent stability through a click reaction (Figure 10). These gels could be used to adhere bacteria without the release of metal ions, ensuring its applicability in the biomedical field. In addition, the polymer films presented better aqueous stability than those of traditional MOFs and they could be stable in the cytoplasmic solution without collapse [148]. The authors demonstrated an innovative method for preparing a porous polymer film, in which the resultant films from the precursor of MOF retained the advantage of their high porosity, improving in the flexibility, facilitating easy to stretch. These advantages would specifically enable the broadening of their industrial application prospects as well. In summary, these highly porous polymer films prepared by using MOF as a precursor has overcome the inherent disadvantages of MOF, and are very promising in biomedical applications.

## 4. Metal-Polymer Ligands

**Figure 11:**
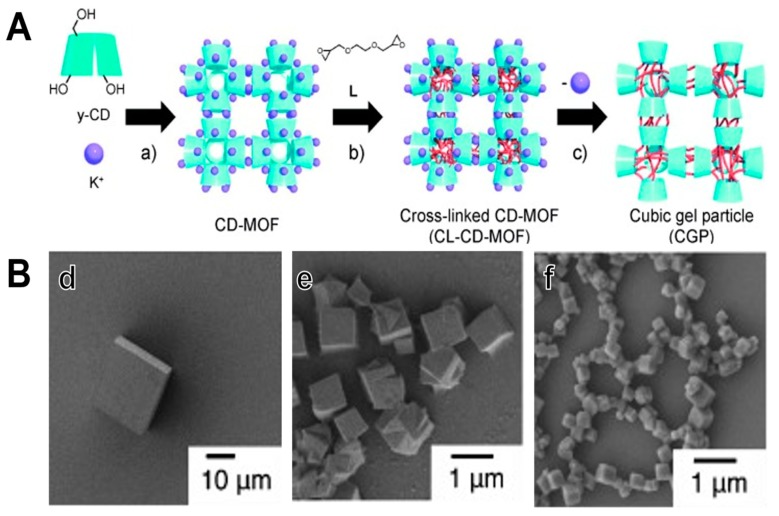
(**A**) Schematic illustrating the synthesis of cubic gel particles. (**a**) Crystallization, (**b**) Cross-linking reaction, and (**c**) Removal of coordinated metal ions. (**B**) SEM images of (**d**) CD-MOF-Micro1, (**e**) CD-MOF-Micro2, and (**f**) CD-MOF-Nano. Reproduced with permission from Ref. [149] Copyright 2012, John Wiley & sons.

Although the method of cross-linking MOF to polymer can conveniently transform MOFs to innovative PGs. However, the size of fabricated PGs is generally large reaching tens to hundreds of micrometers, which might not be optimal for biomedical applications such as drug delivery. Nevertheless, this size enables them to be applied for tissue engineering application. In an attempt to address this issue, Furukawa and colleagues [149], fabricated a variety of polyhedral shape-controllable gel particles using MOF crystals as templates. They initially fabricated polymeric network by controlling the grain size of the MOF and then cross-linked the hydroxyl group and the phenolate group of cyclodextrin (CD) to reduce their solubility in water (Figure 11). It could be observed that by using internal cross-linking of CD-MOF crystals, uniform cubic gel particles with sharp edges and square surfaces were prepared without coordination of metal ions. The cubic gel particles retained the morphology of the original CD-MOF composite crystal, demonstrating that by stringently monitoring the recrystallization conditions, the average of CGPs could be conveniently reduced to nanometer range from millimeters. As CD-MOFs without crosslinking are readily soluble in water, while the cross-linked CD-MOFs were hydrophobic and swollen in water. Cubic gel particles (CGP) upon swelling turned out to be amorphous, indicating that the crystal structure of MOF was destroyed and has changed from a hard material to a soft material. This method significantly opens up new fields for the preparation of micro- and nano-polyhedral polymer gels with appropriate morphology suitable for biomedical applications.

## 5. PolyMOF

**Figure 12:**
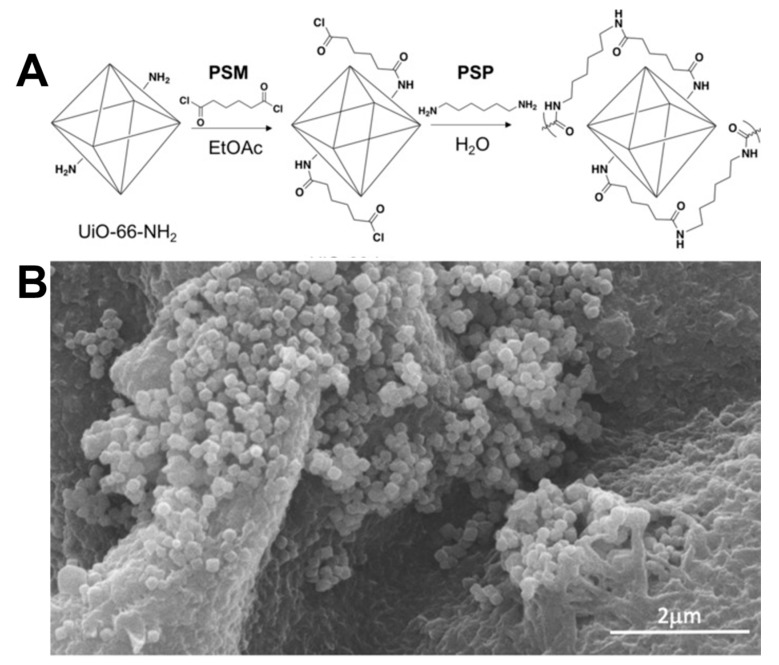
(**A**) Schemes of PSM and PSP used to prepare PA-66-UiO-66-NH_2_. (**B**) SEM image of PA-66-UiO-66-NH_2_. Reproduced with permission from Ref. [152] Copyright 2012, John Wiley & sons.

In addition to coating polymers over the surface of MOFs to improve their physicochemical characteristics, it is feasible to fabricate innovative composites of MOFs using polymeric monomers resulting in the polymer-MOF as a repeating unit as the monomer can endow MOF numerous properties such as chemical protection, decontamination, and hydrophobic property, among others [150,151]. The composite material of MOFs with polymer exhibits the combination of excellent properties of MOFs and polymer. Nevertheless, the fabrication of such MOF-polymer composites (polyMOF) requires a high degree of dispersion and interaction between the MOF particles and the polymer matrix, which remains a significant challenge. In an attempt to solve this issue, Kalaj and colleagues [152] synthesized nylon-MOF by interfacial polymerization technology. During the processing, the surface-modified UiO-66-NH_2_ MOFs were copolymerized with the budding polyamide fiber (PA-66) in the aqueous solution, resulting in PA-66-UiO-66-NH_2_ (Figure 12).

**Figure 13:**
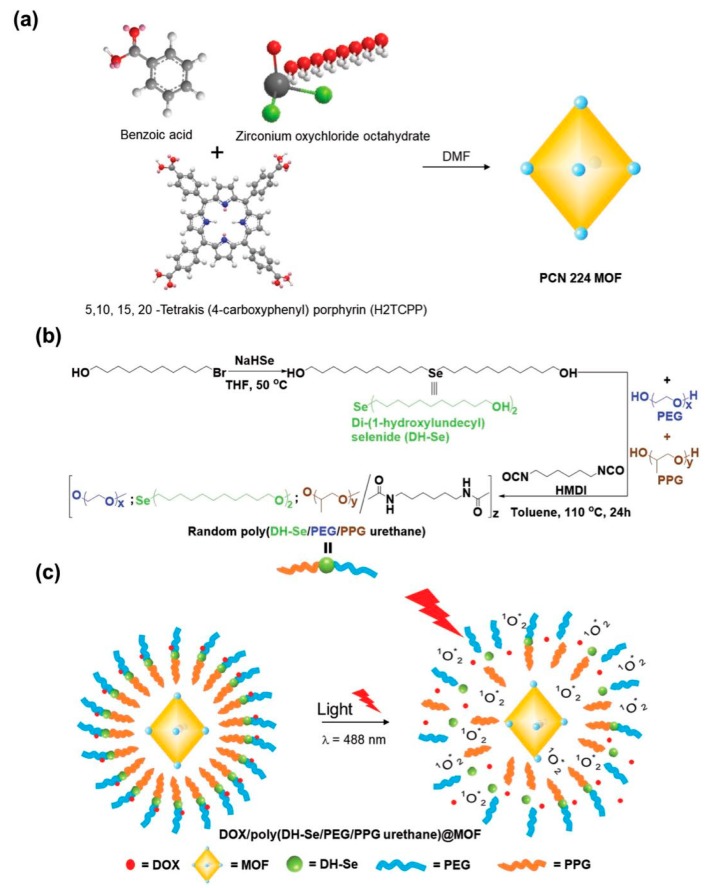
Schematic illustration of the synthesis of porphyrin PCN-224 MOF. (**a**) selenium containing random copolymer poly(DH-Se/PEG/PPG urethane) (**b**) and the design of proposed core/shell nanocomposite by using PCN-224 MOF and poly(DH-Se/PEG/PPG urethane) (**c**) whose structure could be broken with 488 nm light illumination and release the encapsulated anticancer drug DOX for precise chemotherapy. Reproduced with permission from Ref. [153] Copyright 2019, John Wiley & sons.

In general, the chemotherapeutic drugs do not specifically recognize tumor cells, resulting in damage to normal cells while killing cancer cells. For example, DOX is one of such agents binds to the mitochondrial DNA of the cell upon internalization, producing Reactive oxygen species (ROS), which are highly cytotoxic and cause apoptosis. However, whether in normal cells or cancer cells, DOX is capable of producing ROS, which inevitably cause damage to the cells that internalized DOX. Luo and coworkers [153] designed a rational drug controlled release system, which is of great significance for cancer treatment (Figure 13). The core-shell nanoparticle-based controlled drug delivery system using di-(1-hydroxylundecyl) selenide (DH-Se), poly(ethylene glycol) (PEG), and poly(propylene glycol) (PPG) as polymers and Zr-TCPP as a core was fabricated. Zr salt was selected as Zr-MOFs could result in excellent stability in the aqueous solution, without any signs of collapse of porous architectures of MOFs. On the other hand, TCPP was selected as the organic ligand because TCPP, a photosensitive agent, could efficiently ablate tumor cells upon light irradiation at a specific wavelength through producing ^1^O_2_. In addition, the surface-coated PEG not only enhanced their stability in the aqueous environment but also extended their long-term circulation in blood. Interestingly, the polymers were selected in a way to exhibit the synergistic effects in protecting the eventual formulation by offering stability and exhibiting the therapeutic effects. The outer hydrophilic PEG layer extended the circulation time, while the inner hydrophobic PPG layer prevented the surrounding water into the interior of the nanoparticle, avoiding the premature drug release and collapse of MOFs. The generation of deadly ^1^O_2_ from O_2_ by TCPP in the presence of light and strong redox responsiveness of Se, facilitated the decomposition of the outer shell, resulting in the controlled release of drugs. This is the first time that MOF and selenium-substituted polymers have been used as controllable drug release carriers, which may contribute to the combination of precision chemotherapy and photodynamic therapy.

## 6. Characterization of Polymer-Coated MOFs

**Figure 14:**
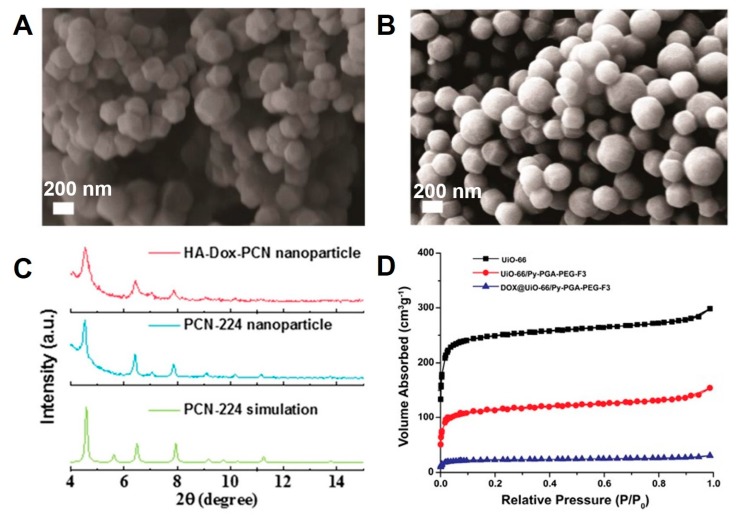
SEM images of (**A**) UiO-66-L2 and (**B**) UiO-66-L2-PNIPAM. Reproduced with permission from Ref. [142] Copyright 2018, American Chemical Society. (**C**) PXRD pattern of PCN-224, HA-Dox-PCN-224. Reproduced with permission from Ref. [126] Copyright 2019, American Chemical Society. (**D**) Nitrogen adsorption-desorption isotherms of UiO-66, UiO-66/Py-PGA-PEG-F3, and DOX-loaded UiO-66/Py-PGA-PEG-F3. Reproduced with permission from Ref. [140] Copyright 2017, American Chemical Society.

More often, various characterization techniques can be used to confirm substantial modifications of polymeric coatings made on the surfaces of MOFs. There are several predominantly used approaches for determining various morphological and physicochemical attributes of MOFs and their subsequent polymeric-coated composites. In this section, a brief account on the predominantly used characterization techniques is given. Predominantly, the morphological attributes are often observed using SEM and TEM recordings, which give an explicit view on the overall particle diameter as well as the shape of the MOFs as well as the polymer-coated MOFs. In addition, they would also provide information of stability attributes such as aggregation of MOFs. In general, MOFs possess different crystal faces due to variation in the crystal growth rates, resulting in diverse morphologies such as different shaped crystalline architectures such as spheres, rods, and cubes, among others. More often, the encapsulation of MOFs in polymer tend to result in the intrinsic shape of MOFs. However, in some cases, owing to the amorphous nature of polymers, the wrapped MOFs tend to be spherical due to the even distribution of polymer over the surface of MOFs. In a case, after encapsulating the MOFs with polymer, the angular edges of MOFs gradually softened and the eventual morphology tended to be spherical [142] (Figure 14A,B). Contrarily, the TEM observations can be used to demonstrate the internal structure as well as the final size of the MOF-polymer composite. In addition to morphology, the physico-chemical attributes can be demonstrated by various techniques. The recording of powder X-ray diffraction (PXRD) patterns is one such approach for determining the crystallographic validations of MOFs. Owing to differences in their composition, the polymers as well as MOFs possess different crystalline forms, which would result in varied crystalline patterns. In some instances, it is feasible to demonstrate the successful encapsulation of MOFs within the polymeric frameworks. In an example, the characteristic PXRD patterns of PCN-224 remained unchanged after coating with HA polymer, indicating that the coating of HA has no influence on the crystallinity of PCN-224 [126] (Figure 14C). However, a slight reduction in the intensities of corresponding peaks would represent the successful encapsulation of MOFs in the polymer. Owing to their high porosity, large specific surface area, and ability of adsorption as well as retention of gases, it can be feasible to evaluate the morphological attributes of MOFs and the polymer-coated MOFs in terms of porosity using nitrogen (N_2_) adsorption-desorption isotherms. The changes in the porosity and reduction in the specific area due to partial blockage of the pores in MOFs after coating with polymers can be evaluated. In general, the specific surface area of MOFs is around 1000 m^2^/g, which could be significantly reduced after coating with polymers. In a case, UiO-66 MOFs resulted in their surface area of 988.625 m^2^/g, which drastically reduced to 438.978 m^2^/g after coating with the polymeric composite of Py-PGA-PEG-F3 [140] (Figure 14D). Further, various other physicochemical characterization techniques such as thermogravimetric analysis (TGA), solid state nuclear magnetic resonance (NMR), and Fourier transform infrared (FTIR) spectroscopic analyses can also be used to demonstrate the stability as well as chemical functionalities of the MOF-polymer composites.

## 7. Conclusions and Perspectives

In summary, this review gave an overview of the various combinations of MOF and polymer, including coating, cross-linking MOF to polymer, metal-polymer ligands, and polyMOF, with excellent physicochemical attributes toward their applicability in the field of biomedicine. By considering various synthetic strategies, polymers were integrated with MOFs, endowing MOFs numerous fascinating functional attributes and improving their aqueous stability, which could compensate for their natural defects. However, most of the MOFs currently used in the biomedical field are Fe-based MOFs and Zr-based MOFs because of their excellent water stability, and the use of other metal-based MOFs in the biomedical field is rarely reported. 

Although polymer-coated MOFs have shown excellent performance efficiency in improving the colloidal stability biocompatibility of MOFs, these composites still suffer from limitations. The precise control of polymer encapsulation on separate MOFs becomes a significant challenge. At present, the biggest problem is that it is easy to wrap several MOF nanoparticles with a polymer. However, the encapsulation of MOFs in polymers may causes agglomeration of the nanoparticles. With the advent of more coating methods, the encapsulation of the polymer on the MOF will be more uniform and can be more widely used in the diverse biomedical fields. With the deepening of MOF and polymer research, more and more MOFs will be applied in the field of biomedicine, and the application of MOF in biomedical fields will continue to be endorsed.

## Figures and Tables

**Table 1:** Polymer-coated MOFs synthesized using various approaches for diverse advanced biomedical applications.

MOF@Polymer	Organic Linkers	Synthetic Process	Particle Size (nm)		Outcome	Reference
			MOFs	MOF@Polymer		
MIL-100(Fe)@ chitosan	BTC	MAS	135 ± 20	204 ± 32	Improved biocompatibility of oral nanocarriers	[112]
MIL-100(Fe)@ heparin	BTC	MAS	155 ± 61	178 ± 44	Toward stealth drug nanocarriers	[113]
PCN-224@HA	TCPP	STS	164 ±20	250 ± 20	Advanced anticancer therapy	[126]
BSA@ZIF-8@PVP	2-MIM	MCS	53±3.1	10 ± 1.6	Intracellular delivery and endo-lysosomal release of native active proteins	[130]
Zr-UiO-66/Py-PGA-PEG-F3	BDC	STS	220	250	In vivo targeting and positron emission tomography imaging of tumor	[140]
GdMOF@PAA	BDC	STS	155 ± 30 (l) and 30 ± 11 (w)	158 ± 30 (l) and 33 ± 11 (w)	Contrast agents for computed tomography and magnetic resonance bimodal imaging	[141]
UiO-66-L1-PolyLact	BDC	STS	143 ± 31	177 ± 25	Selective anticancer cytotoxicity and immune system response	[142]
UiO-66-L2-PNIPAM	BDC	STS	142 ± 14	177 ± 24	Selective anticancer cytotoxicity and immune system response	[142]

Note: l refers to length, W refers to Width.

## Abbreviations

^1^O_2_	Singlet oxygen
2-MIM	2-methyl imidazole
AuNRs	Gold nanorods
AzM	Azide-tagged MOF
AzTPDC	diazide-triphenyl dicarboxylic acid
BDC	1,4-benzene dicarboxylic acid
BSA	Bovine serum albumin
BTC	1,3,5-benzene tricarboxylic acid
CD	Cyclodextrin
CGP	Cubic gel particles
CL4	Acetylene cross-linker
CL2	Diacetylene cross-linker
CLM	Cross-linked MOF
CTAB	Cetyltrimethylammonium bromide
DA-SBDC	Diazido-stilbenedicarboxylic acid
DH-Se	Di-(1-hydroxylundecyl) selenide
DOX	Doxorubicin
ECM	Extracellular matrix
EDX	Energy dispersive X-ray spectroscopy
FTIR	Fourier transform infrared
HA	Hyaluronic acid
PSS	Poly(sodium 4-styrenesulfonate)
PVP	Poly(N-vinylpyrrolidone)
PXRD	Powder X-ray diffraction
MAS	Microwave-assisted synthesis
MCS	Mechanochemical synthesis
ZIF	Zeolitic imidazolate framework
MOFs	Metal-organic frameworks
MSNs	Mesoporous silica nanoparticles
MTP	MOF-templated polymer
NMR	Nuclear magnetic resonance
PA-66	Polyamide fiber
PAA	Poly(acrylic acid)
PCN	Porous coordination network
PDT	Photodynamic therapy
PEG	Poly(ethylene glycol)
PG	Polymer gel
PNIPAM	Poly-N-isopropylacrylamide
PolyLact	Poly-l-lactide
polyMOF	MOF-polymer composites
PPG	Poly(propylene glycol)
Ppy	Polypyrrole
PSM	Post-synthetic modification
PSP	Post-synthetic polymerization
Py–PGA-PEG-F3	Pyrene-derived polyethylene glycol-F3
ROS	Reactive oxygen species
SBU	Second building unit
SEM	Scanning electron microscope
STS	Solvothermal synthesis
TCPP	Tetrakis(4-carboxyphenyl) porphyrin
TEM	Transmission electron microscope
UCNPs	Upconverting nanoparticles
UiO	University of Oslo
ZIF	Zeolitic imidazolate framework
